# Vaporizable endoskeletal droplets via tunable interfacial melting transitions

**DOI:** 10.1126/sciadv.aaz7188

**Published:** 2020-04-03

**Authors:** Gazendra Shakya, Samuel E. Hoff, Shiyi Wang, Hendrik Heinz, Xiaoyun Ding, Mark A. Borden

**Affiliations:** 1 Department of Mechanical Engineering, University of Colorado, 1111 Engineering Dr., Boulder, CO 80309, USA.; 2 Department of Chemical and Biological Engineering, 596 UCB, Boulder, CO 80309, USA.; 3 Materials Science and Engineering Program, 027 UCB, University of Colorado, Boulder, CO 80309, USA.

## Abstract

Liquid emulsion droplet evaporation is of importance for various sensing and imaging applications. The liquid-to-gas phase transformation is typically triggered thermally or acoustically by low–boiling point liquids, or by inclusion of solid structures that pin the vapor/liquid contact line to facilitate heterogeneous nucleation. However, these approaches lack precise tunability in vaporization behavior. Here, we describe a previously unused approach to control vaporization behavior through an endoskeleton that can melt and blend into the liquid core to either enhance or disrupt cohesive intermolecular forces. This effect is demonstrated using perfluoropentane (C_5_F_12_) droplets encapsulating a fluorocarbon (FC) or hydrocarbon (HC) endoskeleton. FC skeletons inhibit vaporization, whereas HC skeletons trigger vaporization near the rotator melting transition. Our findings highlight the importance of skeletal interfacial mixing for initiating droplet vaporization. Tuning molecular interactions between the endoskeleton and droplet phase is generalizable for achieving emulsion or other secondary phase transitions, in emulsions.

## Teaser

Interfacial melting of an internal solid hydrocarbon “skeletal” phase initiates vaporization of liquid fluorocarbon droplets.

## INTRODUCTION

Vaporizable droplets are a special class of reconfigurable complex emulsions (*1*) that have broad applications in ultrasonics (*2*), microfluidics (*3*), energy storage (*4*), heat transfer (*5*), chemical reactions (*6*), and high-energy particle detection (*7*). The liquid-to-gas phase transition of a liquid emulsion droplet to a gas microbubble leads to a volumetric increase by over two orders of magnitude, along with corresponding changes in the particle’s density, compressibility, heat capacity, and other thermophysical properties. This transformation can be exploited for a myriad of applications. In ultrasonics, for example, a relatively passive liquid droplet can be transformed by vaporization into a highly echogenic and acoustically active particle for imaging and therapy (*8*). On a microfluidic chip, the bubble can facilitate fluid mixing and catalyze chemical reactions. Droplet vaporization can also be used to detect high-energy particles, for example, in the quest to find “dark matter” in the universe (*7*).

Droplet vaporization is a thermodynamic and kinetic phenomenon. Thermodynamically, the droplet should vaporize at the boiling point of the liquid phase so long as there is a heterogeneous surface on which to stabilize a vapor embryo. Without such a surface to pin the vapor/liquid contact line, however, this process may be kinetically inhibited owing to the energy barrier for homogeneous critical vapor embryo nucleation (*9*). The spinodal is the thermodynamic limit of stability at which the droplet spontaneously vaporizes by homogeneous nucleation. On the basis of theoretical and experimental observations, the spinodal temperature is usually taken at 80 to 90% of the critical temperature (*T*_c_). Therefore, tuning the critical temperature, in turn, also tunes the vaporization temperature of the droplet. In designing our droplets, we chose C_5_F_12_ as the vaporizable species mainly because perfluorocarbons are biologically inert materials with relatively high vapor pressure (*10*). The presence of one of the strongest intramolecular covalent bonds (C─F) makes it inert to biological processes, volatile owing to weak intermolecular forces, and hydrophobic. Fluorocarbons (FCs) have thus been used for blood expansion (*10*), acoustic droplet vaporization (*11*), and detection of high-energy particles (*7*).

Recent research on biomedical acoustic droplet vaporization has focused on highly volatile species, such as perfluoropropane (C_3_F_8_) and perfluorobutane (C_4_F_10_), to achieve a spinodal near physiological temperature (*12*), but these lighter FCs are more water soluble and therefore rapidly clear from circulation, which limits their utility. Replacing C_4_F_10_ with C_5_F_12_ should notably increase the circulation persistence (*13*), but the latter can be difficult to vaporize (*12*).

Because of the higher spinodal of C_5_F_12_ and accompanying large mechanical index for acoustic droplet vaporization, researchers have focused on heterogeneous nucleation by solid nanoparticle inclusions as a mechanism to effect vaporization (*14*). Our research was inspired by this approach, as well as a recent work on hydrocarbon (HC)/HC endoskeletal droplets (*15*), in which a liquid droplet encapsulates a solid phase. The solid phase provides elasticity to enable nonspherical shapes (*16*). We initially hypothesized that a solid endoskeleton may also serve as a contact line pinning surface for heterogeneous nucleation, and made novel FC/FC and FC/HC endoskeletal drops to test this hypothesis. However, our results indicate that an alternative mechanism of interfacial melting controls vaporization through fluid mixing, providing new engineering opportunities for phase-change droplets.

## RESULTS AND DISCUSSION

**Fig. 1:**
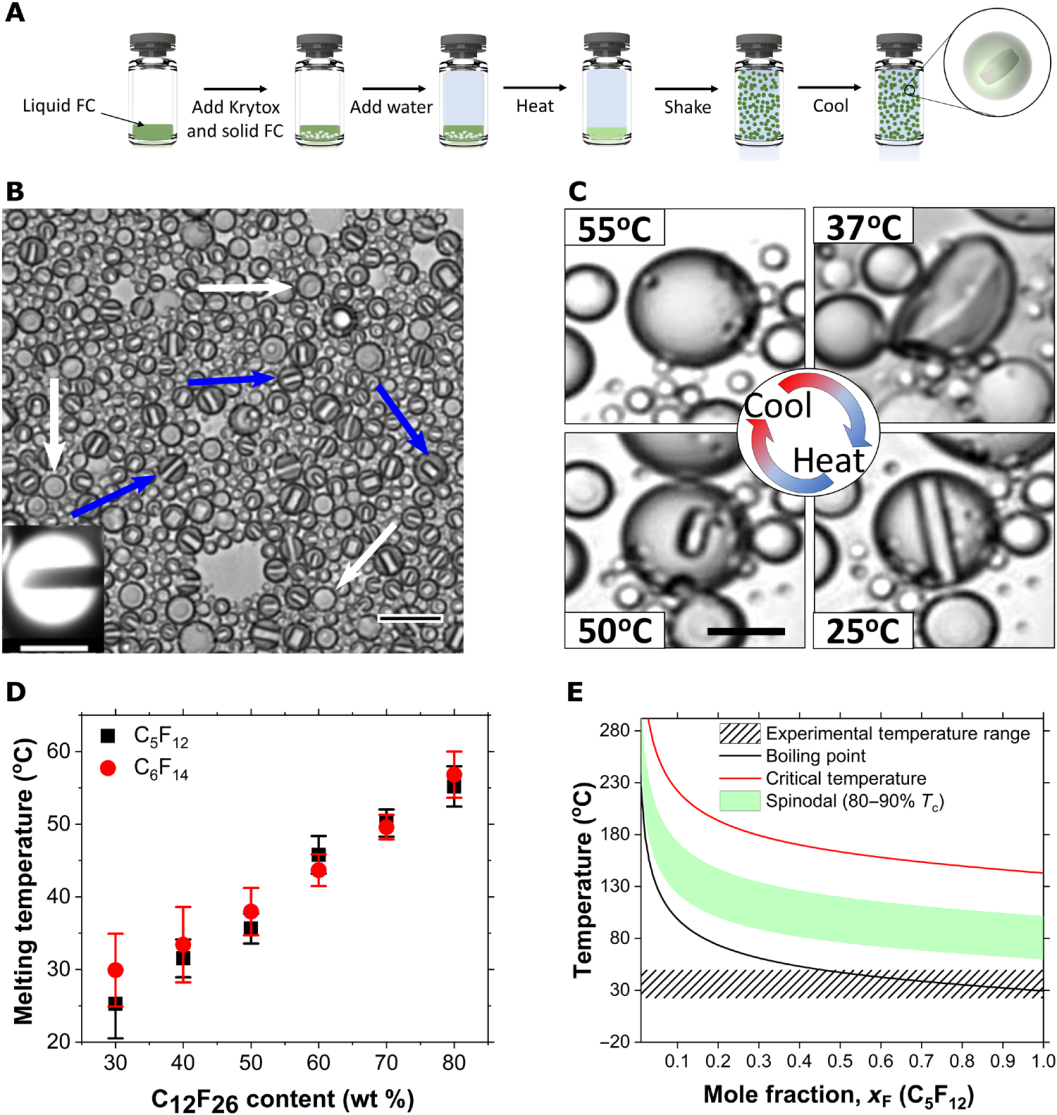
Synthesis and characterization of FC/FC endoskeletal droplets. (**A**) Step-by-step emulsion synthesis process. (**B**) Brightfield microscope image of the endoskeletal droplets showing the unique disc-in-sphere morphology, where C_12_F_26_ forms the solid phase and C_5_F_12_ forms the encapsulating liquid droplet. Blue arrows show side-orientated discs, and white arrows show top-orientated discs; scale bar, 20 μm. Inset shows a fluorescent image with a side-oriented disc; scale bar, 10 μm. The discs were observed to rotate inside the droplets when disturbed by the fluid flow (movie S1). (**C**) At room temperature (25°C), the disc is solid (bottom right). As the droplet is heated, the disc melts (bottom left) and decreases in size until it completely dissolves at a higher temperature (top left). When cooled, the same droplet solidifies by going through a nonspherical phase (top right) and finally forming the sold disc inside the droplet; scale bar, 10 μm. (**D**) Diagram of *T*_m_ versus C_12_F_26_ content shows that the melting depends on droplet composition for two different liquid volatile species: C_5_F_12_ (filled black squares) and C_6_F_14_ (filled red circles). (**E**) *T*_c_ (red), *T*_s_ (green), and *T*_b_ (black) prediction for a mixture of C_5_F_12_ and C_12_F_26_. Note that the spinodal range and experimental temperature range (patterned black lines) do not overlap for this mixture.

To explore the feasibility of heterogeneous nucleation in C_5_F_12_ droplets, we designed a novel endoskeletal architecture with perfluorododecane (C_12_F_26_) as the solid component. Although solid C_12_F_26_ melts at a higher temperature (75°C) than the boiling point of C_5_F_12_ (29°C) (table S1), a liquid mixture of the two species was obtained over a limited temperature range (30° to 65°C for 30 to 80 weight % C_12_F_26_). The C_5_F_12_/C_12_F_26_ liquid mixture was emulsified and then cooled to generate novel endoskeletal droplets, which contained remarkable solid disc structures (Fig. 1, A and B). The droplets were polydisperse with a mean diameter of 3.25 ± 2.28 μm, and their structure was uniform. Increasing C_12_F_26_ content increased the relative disc size. The discs were observed to rotate inside the droplets when agitated (movie S1). Disc formation was not gradual; as the droplet cooled, it first buckled to a nonspherical shape with an internal network structure and then snapped back into a spherical shape with a smooth disc inside (Fig. 1C and movie S2). The disc-in-sphere geometry is consistent with “frazil ice” formed in a supercooled binary melt of water and salt. In frazil ice, the disc-shaped geometry arises from faster interfacial transport in the radial direction than in the axial direction, where the attachment kinetics are limited by both the removal of latent heat released and rejection of solute at the growing interface (*17*).

To examine the vaporization behavior of these novel endoskeletal droplets, they were monitored while being gradually heated (fig. S2). However, heating these droplets to the boiling point of C_5_F_12_ (29°C) did not lead to vaporization, as would be expected for heterogeneous nucleation. Instead, the solid discs gradually melted at a temperature (*T*_m_) that depended on the ratio of C_12_F_26_ to C_5_F_12_ (Fig. 1D). *T*_m_ was found to increase with increasing C_12_F_26_ solid content, but it was always lower than the melting point of pure C_12_F_26_. This experiment was repeated with perfluorohexane (C_6_F_14_) as the volatile species, with similar results. Moreover, these droplets did not vaporize even when heated up to 75°C.

The vaporization temperature of a liquid is determined by intermolecular interactions between the constituent molecules. This effect can be captured quantitatively in a mixture by the exchange parameter (χ) (*18*), which describes the excess free energy of mixing and includes both enthalpic and entropic contributions. In general, χ has a low value for chemically similar blends and a large value for blends that demix easily. In the case of C_5_F_12_/C_12_F_26_ droplets, C_5_F_12_ is a good solvent for C_12_F_26_ (fig. S3) with a low χ value of 0.37. Hence, the boiling point, critical temperature, and spinodal of C_5_F_12_ increase in the presence of C_12_F_26_ (Fig. 1E), which suppresses vaporization. The use of low-χ endoskeletal melting to enhance cohesion and avoid vaporization may be an important strategy for certain applications, such as thermal energy storage.

Using the same logic to design readily vaporizable droplets, we chose to use HCs (alkanes with carbon chain length of 18 to 24) instead of FCs as the solid phase. HCs and FCs do not mix well, as evidenced by their high χ values (5.3 for C_5_F_12_/C_18_H_38_ to 5.6 for C_5_F_12_/C_24_H_50_ at room temperature). We therefore hypothesized that the disruption of FC-FC interactions due to the presence of HC would enable C_5_F_12_ vaporization near physiological temperature.

**Fig. 2:**
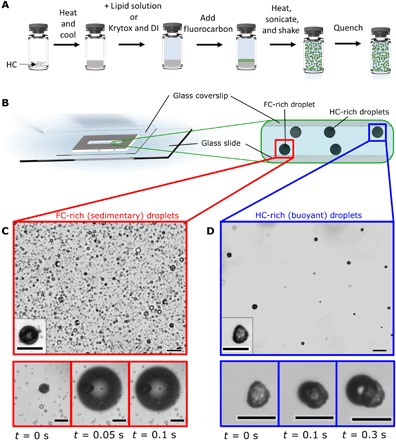
Synthesis and characterization of FC/HC endoskeletal droplets. (**A**) Step-by-step emulsification process with HC as the solid skeletal structure. (**B**) Schematic of the slide setup and side view showing the bimodal density of droplets. (**C** and **D**) Brightfield microscope images of the different droplet species. FC-rich droplets shown in (C) are spherical and sedimentary; hence, they sink to the bottom of the slide. Inset in (C) shows a zoomed-in view of a droplet. Time-lapse images below (C) show the vaporization process of a typical FC-rich droplet. Alternatively, HC-rich droplets seen in (D) are nonspherical in shape and buoyant. Inset in (D) shows a zoomed-in view of a droplet to illustrate the HC skeleton. Time-lapse images below (D) show the vaporization process of the HC-rich droplet. Upon vaporization, the HC skeleton remains attached to the bubble and then slowly spreads over the bubble surface. The more solid HC-rich droplets tended to vaporize more slowly than the more liquid FC-rich droplets (movies S3 and S4). Scale bars, 20 μm for all images.

**Fig. 3:**
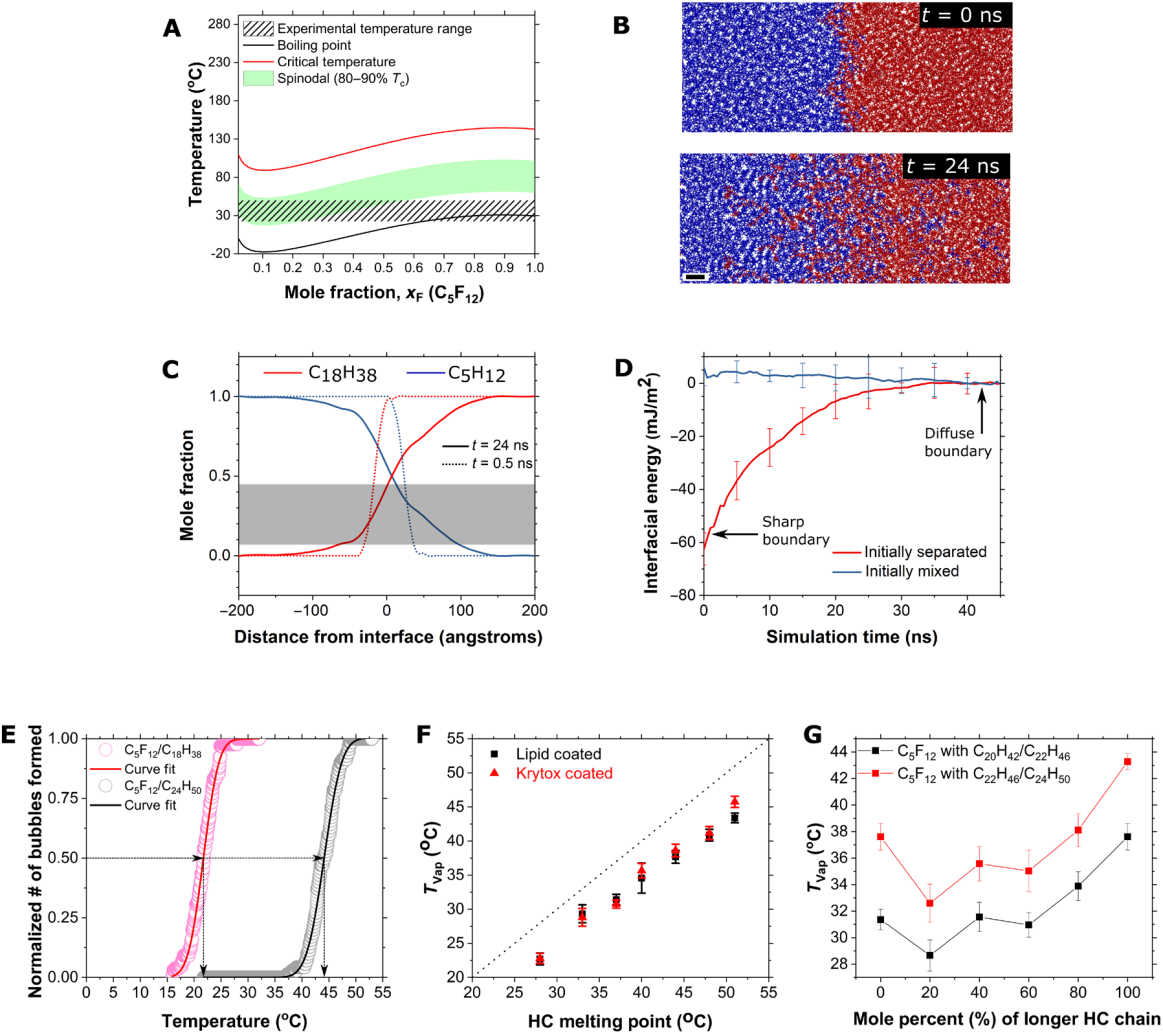
Vaporization properties of FC/HC droplets. (**A**) *T*_c_ (red), *T*_s_ (green), and *T*_b_ (black) prediction for a mixture of C_5_F_12_ and C_18_H_38_. Note that the spinodal range and experimental temperature range (patterned black lines) overlap for this mixture. (**B**) Images from MD simulation of an FC (blue) and HC (red) mixture before (top) and after (bottom) equilibrium, showing the interfacial mixing region. Scale bar, 1 nm. (**C**) Mole fractions of HC and FC in the interfacial region calculated from MD simulation after 0.5 ns (dotted line showing interfacial region before mixing) and after 24 ns (solid line showing interfacial region after mixing) [shown in (B)]. The gray highlight shows the concentration region where vaporization is expected to take place as per (A). (**D**) The interfacial energy during the transition from a sharp phase boundary to equilibrium (red) and the energy of a premixed system (blue) as a function of simulation time. The interfacial energy, given as the total change in inner energy in the interfacial region normalized per cross-sectional area, increases upon mixing, which is consistent with lowering the vaporization energy and the vaporization temperature. Energies are block-averaged every 0.5 ns. The error bars were calculated as the average SD between three blocks centered around the block, where the error is shown for two separate runs of mixed and separated conditions, respectively. (**E**) Typical vaporization curves for C_5_F_12_/C_18_H_38_ droplets (red) and C_5_F_12_/C_24_H_50_ droplets (black). Solid lines represent a normal cumulative distribution function fit. Arrows indicate how the vaporization temperature was calculated from each run. (**F**) Linear dependence of the FC droplet vaporization temperature (mean ± SD) of the droplets versus the HC melting point. Red triangles represent droplets stabilized by a fluorosurfactant (Krytox), and black squares represent droplets stabilized by a hydrocarbon (HC) surfactant (lipids). The black dotted line shows the unity slope with zero offset. (**G**) Where HC/HC mixtures were used for the skeletal phase, the relationship between the droplet vaporization temperature and fraction of longer HC chain was nonlinear owing to the o-d transition to the rotator phase. Black squares represent C_20_H_42_/C_22_H_46_, and red squares represent C_22_H_46_/C_24_H_50_ as the solid HC phase.

FC/HC endoskeletal droplets comprising liquid C_5_F_12_ and solid C_18_H_38_ were prepared in a similar way as the FC/FC droplets (Fig. 2A). However, these droplets had a bimodal morphology owing to differences in HC content. HC-rich droplets were nonspherical and buoyant, whereas FC-rich droplets were spherical and sank to the bottom (Fig. 2, B, C and D). Vaporization was observed in both droplet types at a similar temperature (~22°C), which was lower than the boiling point of pure C_5_F_12_ (Fig. 2, C and D). This observation supported our theoretical prediction that high-χ endoskeletal melting aids in vaporization. In this system, the spinodal is predicted to occur near physiological temperature for C_5_F_12_ concentrations between 5 and 40 mole percent (mol %) in the HC-rich phase (Fig. 3A).

Although the bulk HC and FC liquid phases are immiscible (fig. S4), the interfacial region between them is sufficiently diffuse to allow FC/HC mixing. This was shown with molecular dynamics (MD) simulations (*19*) performed on C_18_H_38_ and C_5_F_12_. The interfacial layer between FC and HC grows to an extension of approximately 10 nm in both directions during the simulation time and then expands no further (Fig. 3, B and C). The interfacial energy increases by about +30 mJ/m^2^ upon mixing in the interfacial region from a sharp interface to a diffuse interface, driven by Brownian motion of the molecules (Fig. 3D). Reduced cohesion after mixing is qualitatively consistent with depression of the vaporization point of C_5_F_12_ observed in our experiments. The formation of a diffuse interface concurs with recent experimental observations of diffuse phase boundaries in atomic resolution (*20*). Previous studies of nucleation and growth have indicated typical initial nucleus sizes of just a few nanometers (*21*, *22*), and the availability of a region of reduced cohesion in excess of 10 nm at the interface of between C_18_H_38_ and C_5_F_12_ supports, in principle, the development of gas bubbles of C_5_F_12_. The time scale of milliseconds for bubble formation in experiments (Fig. 2, C and D) is consistent with a time scale of nanoseconds to microseconds to reform depleted interfaces according to the MD simulation (Fig. 3, B to D).

The robustness of the high-χ endoskeletal melting effect on vaporization was demonstrated experimentally over a homologous series of HC species. Here, the vaporization temperature (*T*_vap_) was defined as the point at which 50% of the droplets vaporized (Fig. 3E). The droplets were observed to vaporize slightly below *T*_m_ of the pure HC phase (Fig. 3F), independent of the surfactant type (HC lipid or FC Krytox) used. Long-chain HCs are known to transition from an ordered phase to a disordered “rotator” phase (termed o-d transition) at temperatures below the actual melting point (*23*). These rotator phases are present in HC with carbon chain lengths of >7 for odds and >20 for evens (*24*). The o-d transition is characterized by the formation of kinks in the HC chains, which make the HC solid phase more liquid-like (*23*). These rotator phases are also responsible for crystallization of nonspherical solids upon controlled cooling of long-chain HC emulsion droplets (*25*). The HC o-d transition temperatures (*26*) correlate well with our experimental vaporization temperatures, indicating that the HC rotator phase facilitates mixing between HC and FC molecules and promotes droplet vaporization.

More evidence for the effect of the HC o-d transition on droplet vaporization was demonstrated, with endoskeletons comprising the HC/HC mixtures eicosane/docosane (C_20_H_42_/C_22_H_46_) and docosane/tetracosane (C_22_H_46_/C_24_H_50_). Phase diagrams of these mixtures show a lowered o-d transition temperature for the mixtures than the pure components (*23*). Corresponding with the phase diagram, C_5_F_12_ endoskeletal droplets formulated with these HC mixtures exhibited a lower vaporization temperature than droplets made with pure components (Fig. 3G). In addition, the range of vaporization temperatures increased with the area of the rotator phase on the phase diagram (fig. S5, C and D). These results support the concept that the o-d transition to the rotator phase in the HC endoskeleton is responsible for vaporization of the liquid FC phase.

**Fig. 4:**
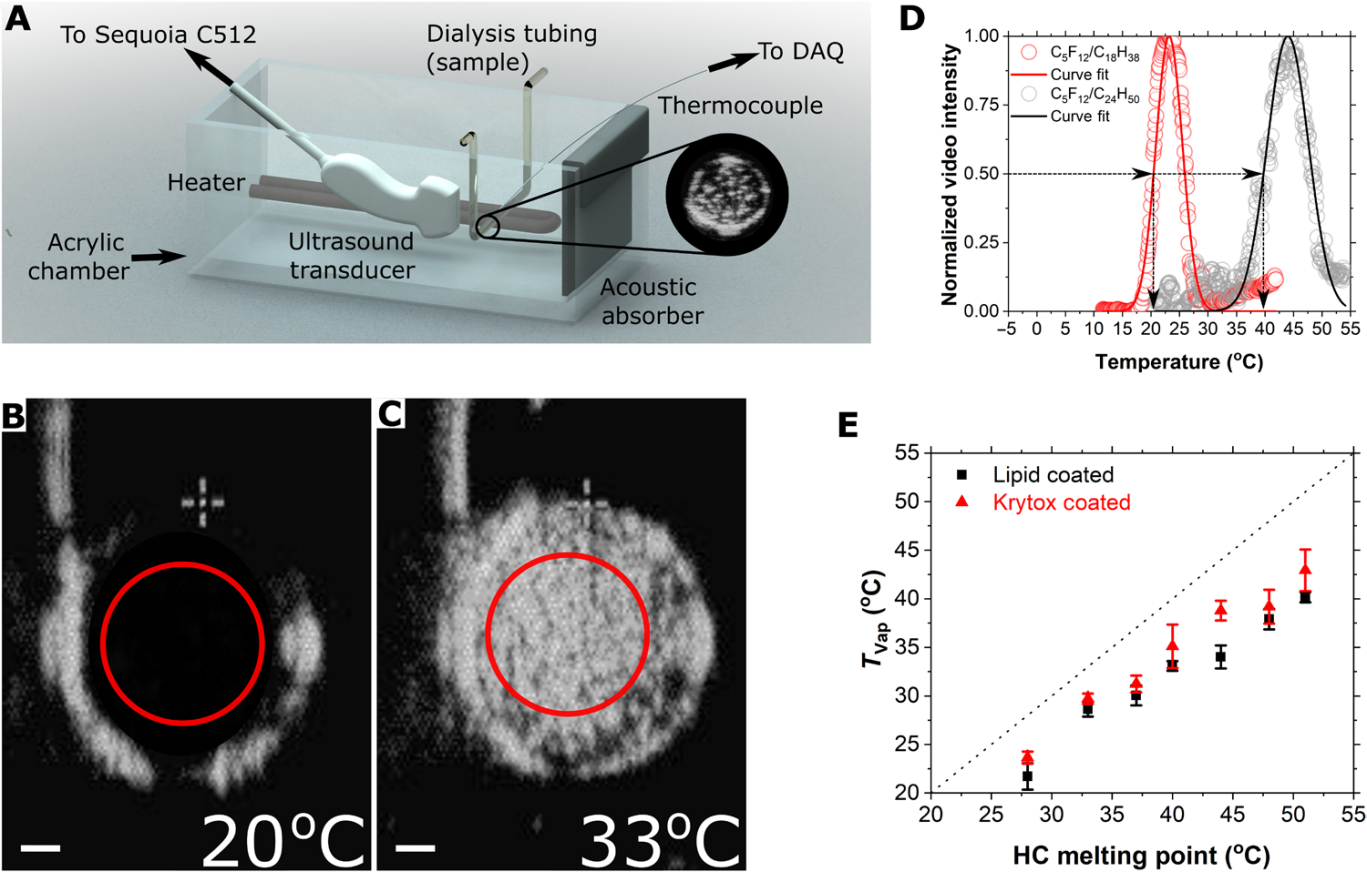
Acoustic verification of the linear dependence of FC droplet vaporization temperature with HC skeleton melting point. (**A**) Setup used for ultrasound experiments. (**B** and **C**) B-mode image of the cross section of a tube filled with endoskeletal droplets at different temperatures. Ultrasound waves are travelling from right to left. Red circles represent the ROI selected for image analysis. The bright vertical line on the top left is the thermocouple. The brighter image in (C) is due to echogenic bubbles formed by droplet vaporization at the higher temperature. Scale bar, 1 mm. (**D**) Typical video intensity curves for C_5_F_12_/C_18_H_38_ droplets (red) and C_5_F_12_/C_24_H_50_ droplets (black). Solid line represents a Gaussian fit done on the data points. Arrows denote how the vaporization temperature was estimated. (**E**) Linear dependence of the FC droplet vaporization temperature (mean ± SD) with the HC melting point. Red triangles represent droplets stabilized by a fluorosurfactant (Krytox), and black squares represent droplets stabilized by an HC surfactant (lipids).

To demonstrate the utility of these droplets, we incorporated a clinical ultrasound scanner as an imaging source to observe vaporization. The endoskeletal droplets, made with C_5_F_12_ and pure HC, were diluted in water and held in an acoustically transparent dialysis tube. The tube was submerged in a water bath to act as an acoustic coupling and heated with an immersion heater (Fig. 4A). B-mode images showed the cross section of the tube before and after vaporization of the droplets, respectively (Fig. 4, B and C). The red circle denotes the region of interest (ROI) selected to calculate the video intensity, which was used to quantify vaporization. Following the optical experiments, endoskeletal droplets with different HC species were used with either HC or FC surfactants. Temperature at the 50% maximum intensity was taken as the vaporization temperature (Fig. 4D). The results mirrored those of the optical experiments: A linear relationship was observed between the droplet vaporization temperature and the melting point of the pure HC (Fig. 4E). The vaporization temperatures measured by ultrasound were slightly lower (~2°C) than those measured optically, likely due to acoustic effects.

These endoskeletal FC droplets with FC or HC solid cores thus provide a previously unknown method of controlling thermal stability for new and existing applications of emulsion droplet vaporization. Use of the low-χ FC endoskeleton stabilizes the liquid phase, whereas the high-χ HC endoskeleton facilitates vaporization. For the latter, nucleation of the vapor phase likely occurs at the FC/HC interface after the solid ordered HC phase transitions to the solid disordered rotator phase. The interfacial melting mechanism is fundamentally different than contact line pinning, solid confinement, and osmotic pressure mechanisms described in previous studies (*27*, *28*). The synthesis method is relatively simple, and the mechanisms described are robust and independent of the surfactant types used in this study. The principle of interfacial mixing, manipulating intermolecular forces, and tuning the spinodal can be broadly applied to various materials, well beyond the initial demonstrations described here. Droplets that do not rely on heterogeneous nucleation could be used, for example, for improving cancer detection, delivering drugs and genes, aiding microfluidic mixing, detecting subatomic particles, or initiating reaction schemes in temperature-sensitive microreactors. Moreover, the linear dependence on melting point and the acoustic imaging capability of the post-vaporization bubbles could also be exploited as a means for a nondestructive in situ thermal probe in high scattering media. The ability to tune the thermodynamic limit of stability for endoskeletal emulsions by interfacial melting will likely find abundant applications.

## MATERIALS AND METHODS

### Materials

The following chemicals were used as received: perfluoropentane (C_5_F_12_, 98%, Strem Chemicals, Newburyport, MA, USA); perfluorohexane (C_6_F_14_, 99%, FluoroMed, Round Rock, TX, USA); perfluorododecane (>99%, Fluoryx Labs, Carson City, NV, USA); Krytox 157 FSH oil (Miller-Stephenson Chemicals, Danbury, CT, USA); 1,2-dibehenoyl-*sn*-glycero-3-phosphocholine (DBPC) (99%, Avanti Polar Lipids, Alabaster, AL, USA); *N*-(methylpolyoxyethylene oxycarbonyl)-1,2-distearoyl-*sn*-glycero-3-phosphoethanolamine (DSPE-PEG5K) (NOF America, White Plains, NY, USA); octadecane (99%), heneicosane (98%), tricosane (99%), tetracosane (99%), and chloroform (≥99.9%) (Sigma-Aldrich, St. Louis, MO, USA); nonadecane (99%, Acros Organics, NJ, USA); eicosane (99%, Alfa Aesar, Ward Hill, MA, USA); docosane (98%, TCI, Portland, OR, USA); DiO fluorescent probe (excitation, 484 nm; emission, 501 nm) (Invitrogen, Eugene, OR, USA); and ultrapure deionized (DI) water from Millipore Direct-Q (Millipore Sigma, St. Louis, MO, USA).

### Preparation of the fluorosurfactant (Krytox) solution

The fluorosurfactant Krytox was mixed to a concentration of 0.75% (v/v) with the FC liquid (C_5_F_12_ or C_6_F_14_, and C_12_F_26_) before adding in other components, such as water or HC.

### Preparation of the HC surfactant (lipid) solution

The lipid solution was formulated by suspending DBPC and DSPE-PEG5K (9:1 molar ratio) at a total lipid concentration of 2 mg/ml in DI water. The lipids were first dissolved and mixed in chloroform in a glass vial, and then the solvent was removed to yield a dry lipid film at 35°C and under vacuum overnight. The dry lipid film was rehydrated using DI water and then sonicated at 75°C at low power (3/10) for 10 min to convert the multilamellar vesicles to unilamellar liposomes.

### Synthesis of FC/FC endoskeletal droplets

The general reaction scheme for synthesizing the FC liquid and FC solid endoskeletal droplet emulsion is shown in Fig. 1A. The solid (perfluorododecane, C_12_F_26_) and liquid (perfluoropentane, C_5_F_12_) FCs were mixed with the fluorosurfactant Krytox [0.75% (v/v) of solution] and DI water, sealed in a glass vial, and heated in a water bath to 30° to 65°C (depending on solid content) until all the solids melted. This heated mixture was then emulsified using a dental amalgamator (TPC D-650 digital amalgamator, 4400 rpm) for 45 s. Depending on the content of the solid, the emulsion was quenched in either an ice bath for droplets containing 50% (w/w) solid content or less, or room temperature water for droplets containing more than 50% (w/w) content. Perfluorohexane (C_6_F_14_) liquid droplets were prepared in the same way as described above.

### Fluorescent labeling of FC/FC endoskeletal droplets

Fluorescently labeled FC/FC droplets were synthesized (using Krytox as surfactant) as above. Fluorescent dye (5 μl/ml; DiO) was added to solid/liquid FC mixture before heating the mixture. DiO was observed to dissolve into the FC liquid phase, but it was excluded from the FC solid phase.

### Synthesis of FC/HC endoskeletal droplets

#### *Endoskeleton made from pure HC*

The general reaction scheme for synthesizing FC/HC endoskeletal droplets is shown in Fig. 2A. The solid HC was weighed (60 mg) in a glass vial and then heated in a water bath to a temperature that was 3°C above the melting point of the HC used (table S1). This was done to prevent HC crystals from dispersing into the aqueous liquid before emulsification. Then, the HC phase was quenched in an ice water bath to form a solid film. For emulsion stabilized by the fluorosurfactant, 0.75% (v/v) Krytox (30 μl) was added and then the aqueous phase (4 ml) was chilled in ice water bath. For emulsion stabilized by the HC surfactant lipid, chilled lipid solution (4 ml) was added to the quenched HC film. Then, 200 μl of C_5_F_12_ was pipetted into the HC/aqueous mixture in the glass vial. The vial was then sealed with a crimper (Wheaton, Millville, NJ, USA), heated to 3°C above the melting point of the HC used, and bath-sonicated for 1 min at 240 W to presuspend the liquid FC and HC phases. This mixture was then emulsified using the amalgamator. The resulting emulsion was quenched in ice water to form the final FC/HC endoskeletal droplets.

#### *Endoskeleton made from a mixture of HCs*

The required ratios of different HCs (20, 40, 60, and 80 mol % of C_22_H_46_ in C_20_H_42_ or C_24_H_50_ in C_22_H_46_) were weighed (to make a total of 60 mg) in a glass vial and then heated in a water bath at a temperature that was 10°C higher than the melting point of the higher chain length HC (55°C for C_20_H_42_/C_22_H_46_ mixture and 60°C for C_22_H_46_/C_24_H_50_ mixture). This was then quenched in an ice water bath to form a solid film at the bottom of the vial. The procedure for synthesizing FC/HC endoskeletal droplets was then used as described above. Only the HC surfactant lipids were used for these endoskeletal droplets.

### Sizing and counting the droplets

Droplet size and concentration were measured using an Accusizer 780A counter (PSS Nicomp, Port Richey, FL, USA), which sizes individual particles as they pass by a laser using forward and side scattering.

### Optical heating experiments

The optical heating experimental setup (fig. S2) consisted of a glass slide (25.4 mm × 76 mm, Fisher Scientific) heated by two flexible heaters (Kapton KHLV-102/10-P, Omega Engineering, Norwalk, CT, USA). The heaters were attached to a power supply (Agilent E3640A, Agilent Technologies, Santa Clara, CA, USA). The sample was diluted by 3:7 with DI water and pipetted (100 μl) into the well of a custom microscope chamber. A spacer with a well was made by three-dimensional (3D) printing (Stratasis Objet30, Eden Prairie, MN, USA) with two holes for K-type thermocouples (Omega Engineering 5TC-TT-K-36-36, Norwalk, CT, USA). The 3D-printed spacer was sandwiched between a glass slide and coverslip (24 mm × 50 mm, Fisher Scientific) using a thin film of vacuum grease (Dow Corning, Houston, TX, USA). A proportional-integral-differential (PID) controller was built and implemented to control the temperature and temperature rise rate of the chamber. The chamber was attached to an inverted microscope (Nikon Eclipse Ti2 Inverted Microscope, Melville, NY, USA) fitted with Nikon Plan Fluor 4× and 10× objectives. The microscope was attached to a digital complementary metal-oxide semiconductor (CMOS) camera (Hamamatsu C11450 ORCA Flash-4.0LT, Bridgewater, NJ, USA). Temperature points were collected using a NI-9212 data acquisition system attached to an NI-TB-9212 isothermal terminal block and run with a custom-built LabVIEW program (National Instruments, Austin, TX, USA) to acquire and store data on the computer (microscope images with a time and temperature stamp) and to control the heater. One thermocouple was used to record the temperature of the sample near the heater, and the other was used to record the temperature of the sample at the center between the two heaters. The thermocouple measuring the temperature of the sample close to the heater was set as the controlled variable owing to the faster time constant and hence greater controller stability. The thermocouple used to measure the temperature at the center between the heaters was considered to be the true sample temperature. The typical difference was about 2° to 3°C between the center and edge of the sample holder. The microscope stage was translated to find a field of view with 2 to 15 droplets close to the center thermocouple. Image acquisition and data collection started when the PID controller was turned on.

#### *FC/FC endoskeletal droplets*

For FC/FC droplets, the sample was slowly heated from room temperature until all the solid disc structures inside the droplets melted. Then, the heater was turned off as the sample was allowed to cool slowly under ambient conditions back to room temperature. For each composition, three to four samples were synthesized, and three to four separate heating runs were performed per sample (*n* > 20 droplets per composition).

#### *FC/HC endoskeletal droplets*

For FC/HC droplets, the sample was slowly heated from room temperature to 50°C. Images were captured at a rate of 5 frames/s. Vaporization was observed by conversion of the semitransparent drop to a larger, high-contrast bubble. The number of bubbles was counted in each frame and coded to the corresponding time and temperature. The normalized number of bubbles (normalized to 1 by dividing by total maximum number of bubbles formed at the end of the run) was plotted against the sample temperature (Fig. 3D). A normal cumulative distribution function was fit to the data using OriginPro (OriginLab, Northampton, MA, USA). The temperature corresponding to 50% vaporization from the fit was selected as *T*_vap_ (vaporization temperature) for the droplet sample. This process was repeated at least three times per sample for at least three separately prepared samples per composition. Hence, at least nine plots were formed for each FC/HC mixture. The mean and SD for *T*_vap_ is plotted in Fig. 3E for each composition. The same process was repeated for all the compositions and for the different surfactant coatings. This procedure was done for both pure HC and mixed HC droplets.

### MD simulations of FC/HC interface

Models of perfluoropentane (C_5_F_12_) molecules and octadecane (C_18_H_38_) molecules were prepared in all-atom resolution using the Materials Studio program. Two simulation boxes containing 1890 C_5_F_12_ molecules and 1062 C_18_H_38_ molecules, respectively, were pre-equilibrated for 20 and 10 ns, respectively, until they reached bulk density and equilibrium. To explore the interfacial properties, the two bulk components were then combined with a 15.7-Å-thick platinum slab added to the bottom of the simulation box to avoid periodic interactions between the two components. The final simulation box was at a size of 43.2 × 43.2 × 666.6 Å^3^, which was large enough to represent bulk properties and observe interfacial behavior. Simulations of the final simulation box were run for 18 ns when the system reached equilibrium (Fig. 3B). Density profiles of the C_5_F_12_ and C_18_H_38_ molecules were calculated from the last 3 ns of the simulation, and the first 1 ns, and used to create a plot of mole fraction of each component against distance at the material interface. The raw mole fraction data were smoothed with a third-order polynomial using the Savitzky-Golay method (Fig. 3C).

A smaller simulation was run to obtain energy values of a system of HC and FC as it mixes. The system contained 630 C_5_F_12_ and 354 C_18_H_38_ molecules to keep the same ratio as the previous simulation. FC and HC were fully separated initially. Systems were run for over 50 ns to equilibrium, and energies were calculated using 0.5-ns block averages for the next 45 ns, with the equilibrium energy of the premixed system set as the 0 energy reference point. A smoothing function was applied to both curves, and energy values were converted from kcal/mol to mJ/m^2^ based on the cross-sectional area of the initially separated FC/HC simulation cell (42.36 × 42.36 Å). This was done twice for both the mixed and separated systems.

MD simulations were carried out in the NPT (isobaric-isothermal ensemble) ensemble using the LAMMPS (large-scale atomic/molecular massively parallel simulator) program and the PCFF (polymer consistent force field) force field (*29*, *30*). The time step was 1 fs, the summation of Lennard-Jones interactions included a cutoff of 1.2 nm, and the summation of electrostatic interactions was carried out in high accuracy (10–^5^) using the PPPM (particle-particle particle-mesh) method. Temperature and pressure were maintained at 308.15 K and 1 atm to match experimental conditions.

### Ultrasound heating experiment setup

The ultrasound experimental setup (Fig. 4A) consisted of a custom-built acrylic chamber. The temperature of the water bath was controlled by an immersion heater (Heat-O-Matic 335, 115 V, 500 W, Cole-Palmer, Vernon Hills, IL, USA). The sample was pumped through dialysis tubing (6.37-mm dry diameter, Fisher Scientific), which was fully submerged in the water bath. The chamber consisted of two magnetic stirrers and sat atop two magnetic stir plates while continuously stirring the water bath for uniform heating. A magnetic stirrer was also placed inside the dialysis tubing to ensure that the sample was continuously mixed. A k-type thermocouple (Omega Engineering 5TC-TT-K-36-36, Norwalk, CT, USA) was used to measure the temperature of the water bath, with the tip placed close to the sample tubing. A rubber layer was attached to the wall of the chamber to prevent acoustic reflection from the acrylic. Temperature was acquired using NI-9212 DAQ (National Instruments, Austin, TX, USA). Ultrasound images were collected using an ultrasound transducer (Acuson 15 L8, Siemens, Tarrytown, NY, USA) attached to a clinical ultrasound system (Acuson Sequoia C512, Siemens). B-mode images were taken at a frequency of 8 MHz and tissue attenuation–derated mechanical index of 0.29 for all the experiments. The 2D gain was set to 0 dB, and dynamic range was set to 50 dB for all the images taken. Images were acquired using LabVIEW. The droplets were diluted to 10^8^ droplets/ml and injected by syringe into the dialysis tubing. The tubing was clamped on both ends to prevent leakage. An image was acquired every second throughout the heating process. Image analysis was done using ImageJ (National Institutes of Health, USA). An ROI was created inside the tube (Fig. 4, B and C) such that the walls of the tubing do not affect the total video intensity. The total (summed) video intensity inside the ROI was calculated for each frame, normalized to the maximum video intensity achieved in that acquisition, and plotted against time (and hence temperature) for each run. A representative plot is shown in Fig. 4D. The plot obtained was a bell-shaped curve owing to droplet vaporization and subsequent bubble destruction; hence, a Gaussian function was fit to the normalized curve (normalized to unity) as seen in the figure. The temperature at 50% video intensity was chosen to be the vaporization temperature (*T*_vap_). At least three runs were done for each sample, and at least three different samples were prepared for each FC/HC mixture. The mean and SD of all the runs is plotted in Fig. 4E for each composition. The process was repeated for each surfactant (FC and HC).

## Supplementary Material

aaz7188_Movie_S6.avi

aaz7188_Movie_S5.avi

aaz7188_Movie_S4.avi

aaz7188_Movie_S2.avi

aaz7188_Movie_S3.avi

aaz7188_Movie_S1.avi

aaz7188_SM.pdf

## SUPPLEMENTARY MATERIALS

Supplementary material for this article is available at http://advances.sciencemag.org/cgi/content/full/6/14/eaaz7188/DC1
