# Supplementary material for: Vaporizable endoskeletal droplets via tunable interfacial melting transitions
Source: Sci Adv. 2020 Apr 3;6(14):eaaz7188. doi: 10.1126/sciadv.aaz7188 (PMC7124936; doi:10.1126/sciadv.aaz7188)
Supplement: aaz7188_SM.pdf [file aaz7188_SM.pdf]

Science Advances
AAAS

advances.sciencemag.org/cgi/content/full/6/14/eaaz7188/DC1

Supplementary Materials for

Vaporizable endoskeletal droplets via tunable interfacial melting transitions

Gazendra Shakya, Samuel E. Hoff, Shiyi Wang, Hendrik Heinz, Xiaoyun Ding, Mark A. Borden*

*Corresponding author. Email: mark.borden@colorado.edu

Published 3 April 2020, *Sci. Adv.* **6**, eaaz7188 (2020)
DOI: 10.1126/sciadv.aaz7188

The PDF file includes:

Sections S1 to S3
Table S1
Figs. S1 to S4
Legends for movies S1 to S6
References

Other Supplementary Material for this manuscript includes the following:

(available at advances.sciencemag.org/cgi/content/full/6/14/eaaz7188/DC1)

Movies S1 to S6

Table S1. List of Materials and Properties

Chemical Name	Molecular Formula	CAS #	Molecular Weight (g/mol) (*31*)	Density (g/cc) (*31*)	Melting Point (°C)	Boiling point (°C)	Critical Temperature (°C)
Perfluoropentane	C_5F_{12}	678-26-2	288.04 (*32*)	1.63	-125 (*32*)	29.2 (*31*)	147.4 (*32*)
Perfluorohexane	C_6F_{14}	355-42-0	338.042	1.6910	-86.1 (*31*)	57.2 (*31*)	-
Perfluorododecane	$C_{12}F_{26}$	307-59-5	638.0869	1.73	75 (*33*)	-	-
Octadecane	$C_{18}H_{38}$	593-45-3	254.495	0.7768	28.17 (*31*)	-	-
Nonadecane	$C_{19}H_{40}$	629-92-5	268.521	0.7855	31.5 (*31*)	-	-
Eicosane	$C_{20}H_{42}$	112-95-8	282.547	0.7886	36.48 (*31*)	-	-
Heneicosane	$C_{21}H_{44}$	629-94-7	296.574	0.7919	40.4 (*31*)	-	-
Docosane	$C_{22}H_{46}$	629-97-0	310.600	0.7944	43.8 (*31*)	-	-
Tricosane	$C_{23}H_{48}$	638-67-5	324.627	0.7785	47.4 (*31*)	-	-
Tetracosane	$C_{24}H_{50}$	646-31-1	338.65	0.7991	50.3 (*31*)	-	-

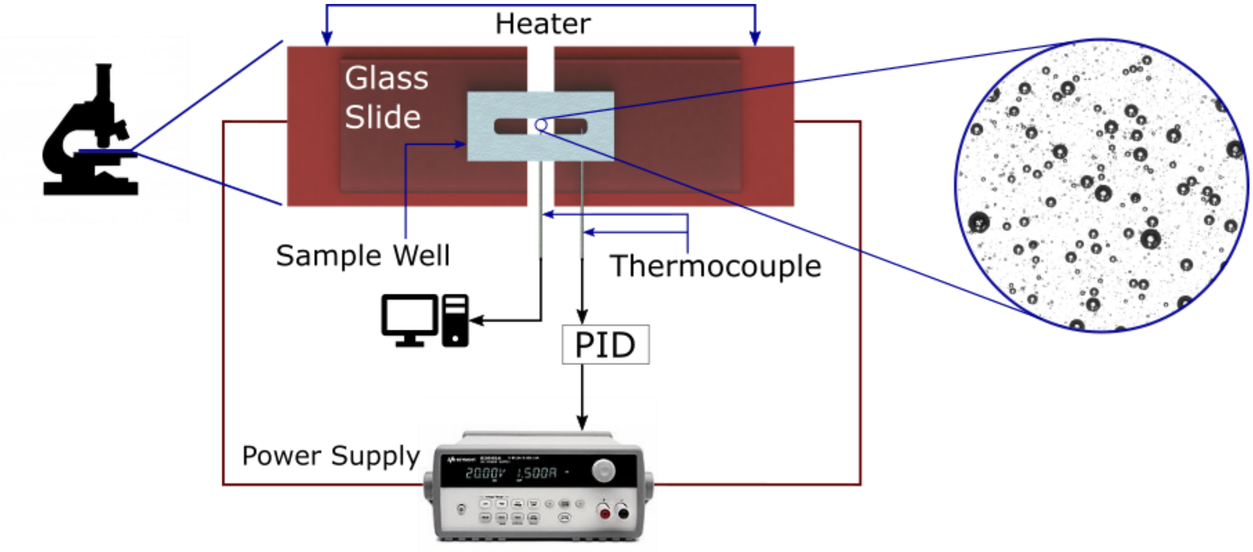

Fig. S1 | Setup used for optical heating experiments. A plastic gasket with a sample well and thermocouple two thermocouple ports is sandwiched between glass slides and mounted onto a microscope stage. A PID controller actuates flexible heaters mounted on the sides to reach the desired temperature. The blowup of the sample shows an example of newly vaporized bubbles.

Section S1. Theoretical Vaporization Behavior of Mixtures

The boiling point of a liquid is defined as the temperature at which the vapor pressure equals the ambient pressure. In the presence of sites for heterogeneous nucleation, such as solid surfaces, vaporization occurs at the boiling point. In the absence of heterogeneous nucleation, vaporization occurs at a higher temperature, and the liquid becomes superheated (*34*). The thermodynamic limit for superheat is called the "spinodal", and it occurs at approximately 80-90% of the critical temperature. Here we analyze the effects of FC/FC and FC/HC mixtures on the vapor pressure, boiling point and spinodal temperature.

Boiling point elevation (or vapor pressure depression) in a binary mixture is a well-established phenomenon. The vapor pressure for a binary mixture can be estimated using the lattice model (*18*). According to the lattice mode, the vapor pressure of a volatile solvent (perfluorocarbon in our case) in a binary mixture at a specific temperature is given by (*18*),

$$P_{vap} = P_0 x_F e^{\chi(1-x_F)^2} \tag{1}$$

where P_{vap} is the vapor pressure of the mixture, P_0 is the vapor pressure of the pure solvent, x_F is the mole fraction of the solvent and χ is the exchange parameter. The exchange parameter (χ) describes the excess free energy of mixing and includes both enthalpic and entropic contributions. It also dictates how ideal the mixture is. For ideal mixtures (χ = 0), the vapor pressure increases linearly with solvent mole fraction and reaches a maximum for the pure solvent. For non-ideal mixtures, vapor pressure depends exponentially on the value of χ. The exchange parameter can be written as a sum of its entropic (χ_s) and enthalpic (χ_H) components,

$$\chi = \chi_H + \chi_s \tag{2}$$

The entropic component of the interaction parameter was set at 0.34 because the FC and HC species are nonpolar (*35*). The enthalpic component depends on temperature and the affinity of the two components. Hildebrand solubility parameters can be used to calculate the enthalpic contribution of χ using the following equation (*36*),

$$\chi_H = \frac{V_F(\delta_1 - \delta_2)^2}{RT} \tag{3}$$

where V_F is the molar volume of the solvent (perfluoropentane), δ_1 is the solubility parameter of the solvent, δ_2 is the solubility parameter of the solid component (either hydrocarbon or perfluorododecane), R is the universal gas constant and T is absolute temperature. The Hildebrand solubility parameter (δ) is a measure of the self-cohesiveness, and the compatibility of two components is quantified by the difference between these quantities. The quantity δ^2 is called the cohesive energy density ($\overline{U}_0$) as it characterizes the strength of the attractions between the molecules. Similar molecules have similar values for δ. Hence mixing becomes more favorable as the difference between the solubility parameters of the two components decreases. In the case of

perfluorocarbons (*37*), δ for perfluoropentane is 11.3 $MPa^{1/2}$ and perfluorododecane is 12 $MPa^{1/2}$. From the δ values of FC mixtures, it can be seen that these components favor mixing. For HC, the solubility parameter δ was calculated from its cohesive energy density ($\overline{U}_0$). The cohesive energy density can be calculated using the molar cohesive energy (U_0), as shown in the following equation,

$$\delta = \sqrt{\overline{U}_0} = \sqrt{U_0/V} \tag{4}$$

Since long-chain alkanes are nonpolar, and the only intermolecular forces acting on it are dispersion forces, the U_0 of alkanes can be calculated based on the strength of dispersion forces for each CH_2 group (*38*).

The exchange parameter values calculated from equations 2, 3 and 4 are plotted for various mixtures at different temperatures in fig. S2a. For consistency, χ values were taken at room temperature in all of the following calculations. Using room-temperature values for χ, vapor pressure was determined and plotted for different solid components as a function of mole fraction of C_5F_{12}. The vapor pressure elevation/depression for various mixtures calculated from equation 1 are shown in fig. S2b. The resulting vapor pressure values were used to predict the boiling point (T_b) of the solvent, as the boiling point is where vapor pressure equals the ambient pressure (1 atm). Boiling point was thus determined from the Clausius-Clapeyron equation:

$$\frac{1}{T_2} - \frac{1}{T_1} = -\frac{R}{\Delta H_v} \ln\left(\frac{P_2}{P_1}\right) \tag{5}$$

where P_1 is the vapor pressure at temperature T_1, P_2 is the vapor pressure at temperature T_2, and ΔH_v is the heat of vaporization of the solvent. The boiling point (T_b) is thus determined by the following equation,

$$T_b = \frac{\Delta H_v T_r}{\Delta H_v + RT_r \ln\left(P_{vap}\right)} \tag{6}$$

The resulting values for T_b (saturation temperature) are plotted in fig. S2c. The critical temperature (T_c) is the temperature above which a vapor cannot be liquefied at any pressure (*39*). Like the boiling point, this temperature is mainly affected by the intermolecular interactions. Hence, an empirical relation between T_c and T_b was established by using a linear least-squares fit to tabulated literature data (*40*) for FCs ranging from perfluoromethane (CF_4) to perlfuorodecane ($C_{10}F_{22}$)(*32*). Data with the trendline is shown in fig. S2d. This relation is given by ($R^2 = 0.998$):

$$T_c = 69.898 + 1.1443 * T_b \tag{7}$$

Combining equations 1, 6 and 7, gives an empirical relation to calculate T_c for perfluoropentane and an additional solute:

$$T_c = \frac{1.1443 * \Delta H_v \mathrm{T_r}}{\Delta H_v + RT_r \ln\left(P_o x_F e^{\chi(1-x_F)^2}\right)} + 69.898 \quad (8)$$

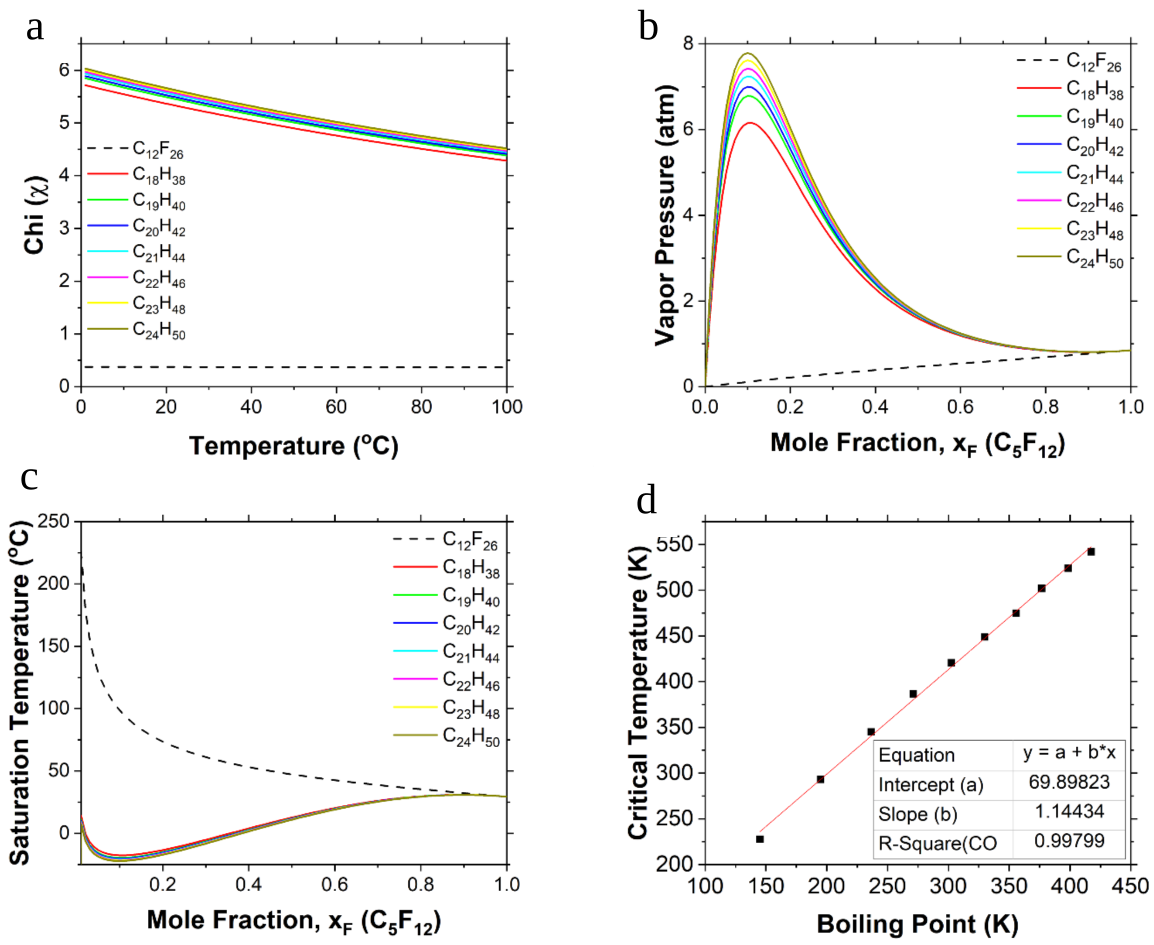

Fig. S2 | Modeling results for different mixtures. a. χ values for mixtures of perfluoropentane (C_5F_{12}) and various materials. χ values are very low for FC/FC mixture compared to FC/HC mixtures. **b.** Consequent values for vapor pressure for different concentrations of C_5F_{12} in the mixture. Vapor pressure elevation can be seen for FC/HC mixtures whereas the vapor pressure decreases for FC/FC mixture. **c.** Consequent values for boiling point for different concentrations of C_5F_{12}. Boiling point depression can be seen for FC/HC mixture whereas it increases for FC/FC mixture. **d.** Empirical relation between the boiling point and the critical temperature of various straight chain fluorocarbons from perfluoromethane (bp 145 K) to perlfuorodecane (bp 417 K).

Experimentally, the spinodal temperature (T_s) is observed to be at 80% to 90% of T_c (*34, 41*). Figure 1e and 3a shows the theoretical predictions of the vaporization temperature, critical temperature and spinodal range for a mixture of perfluoropentane with perfluorododecane and octadecane, respectively. Note that the spinodal temperature range does not drop to the experimental temperature range for FC/FC mixtures, whereas it crosses the experimental temperature range for FC/HC mixtures, thereby demonstrating the effects of intermolecular interactions as captured by the exchange parameter (χ). Low-

χ mixtures (e.g., FC/FC) lead to enhanced cohesive intermolecular interactions, which in turn lower the vapor pressure and raise the boiling point and spinodal temperature. Conversely, High-χ mixtures (e.g., FC/HC) lead to reduced cohesive intermolecular interactions, which in turn increase the vapor pressure and lower the boiling point and spinodal temperature, effecting vaporization.

Section S2. Theoretical Phase Diagram for the $C_5F_{12}/C_{18}H_{38}$ Mixture

The $C_5F_{12}/C_{18}H_{38}$ binary phase diagram was constructed following Carey (*34*). The free energy of mixing was calculated using the equation,

$$\frac{\Delta F_{mix}}{NkT} = xlnx + (1-x)\ln(1-x) + \chi x(1-x) \quad (9)$$

Here, χ is the exchange parameter for the $C_5F_{12}/C_{18}H_{38}$ mixture, and x is the mole fraction of C_5F_{12}. The free energy plot was calculated for different temperatures (-25 ^{o}C to 750 ^{o}C in increments of 7 ^{o}C). Each plot has two minima where the system forms separate phases. These plots were then combined to give the phase-transition temperature vs. mole fraction of C_5F_{12}. This plot was inverted to produce the binary phase diagram, as shown in fig. S3.

The phase diagram for $C_5F_{12}/C_{18}H_{38}$ system shows that the Upper Critical Solution Temperature (UCST) for this system is above 600 ^{o}C. At our experimental temperature range, at equilibrium there consist a two-phase region with C_5F_{12} concentrations of ~1 and ~99 mole% in the HC and FC phase respectively. But, these are the concentrations of the bulk at equilibrium. When we look closely at the interface, even with the presence of two-phase region, it was shown from the MD simulations that the HC and FC phase is diffuse, and a range of concentrations exists as shown in figure 3b and 3c.

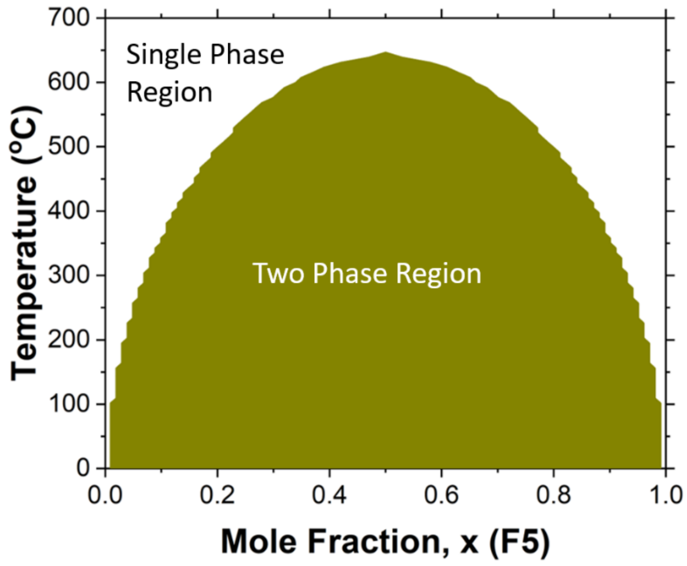

Fig. S3 | Binary Phase Diagram for $C_5F_{12}/C_{18}H_{38}$ mixture. Upper critical solution temperature of >600 ^{o}C is seen for $C_5F_{12}/C_{18}H_{38}$ mixture. At any temperature below that, the mixture separates into two phases.

Section S3. Comparison of Vaporization Temperature and o-d Transition Temperatures

From the experiments, we observed that the endoskeletal droplets with pure HC vaporized at temperatures that are consistently a few degrees below the melting point of the HC used (Figure 3f). In long-chain HCs, rotator phases (solid order-to-disorder transition) are also seen a few degrees below the melting point. This transition is characterized by the formation of many defects in the long HC chain. Hence, after the o-d transition, there is a sudden increase in entropy, abrupt increase in volume, and more liquid-like behavior in

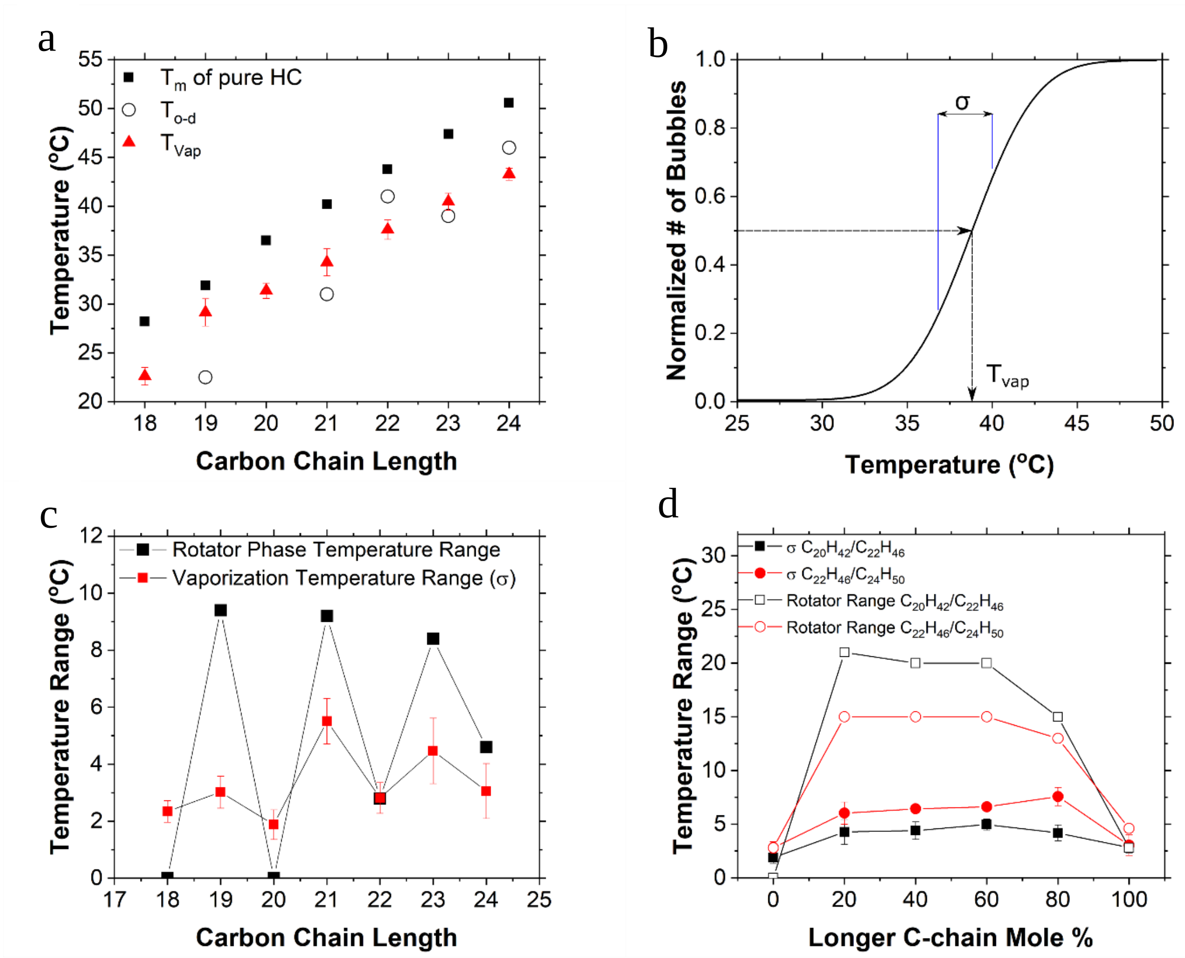

Fig. S4 | Comparing vaporization temperatures with o-d transition temperatures and rotator phases. a. Plot showing the melting point (black solid squares), o-d transition temperature (black blank circles) and the vaporization temperature of endoskeletal droplets (red filled triangles with error bars denoting one standard deviation). Vaporization is predominant in the rotator phase range. **b.** A plot showing a fitted curve to the data from a single vaporization run from droplets made of $C_5F_{12}/C_{22}H_{46}$. σ is the standard deviation which represents the temperature range over which majority of the vaporization takes place. **c.** Plot comparing rotator phase temperature range (range of temperature over which the rotator phase lasts) for pure HC and the vaporization temperature range for droplets made with FC and that pure HC. Notice similar trends in the rise and fall in the temperature range. **d.** Similar plot comparing rotator phase temperature range for mixture of HC and the vaporization temperature range for droplets made with FC the same mixture of HC (filled red circles represents endoskeletal droplets made with C_5F_{12} and $C_{22}H_{46}/C_{24}H_{50}$ mixture, filled black squares represents endoskeletal droplets made with C_5F_{12} and $C_{20}H_{42}/C_{22}H_{56}$ mixture, empty circles and squares are for HC mixtures only).

general (*26*). This liquid-like behavior of the HC, after transitioning from the ordered solid to the disordered solid state, facilitates mixing between FC and HC molecules and promotes droplet vaporization. Figure S4a shows the melting point (T_m), o-d transition temperature (T_{o-d}) and vaporization temperature (T_{vap}) for endoskeletal FC/HC droplets comprising different chain-length HCs. It can be observed that T_{vap} lies below T_m, in the rotator phase region. Increasing the temperature increases the extent of superheat for the liquid C_5F_{12} as well, which might be the reason why endoskeletal droplets with longer chain HCs vaporize easily compared to droplets with shorter chain HCs.

Interestingly, alkanes with even or odd numbered carbon lengths show differences in the range of temperatures over which the rotator phase exists. This temperature range was quantified as the difference between the solid-liquid transition temperature (melting point) and the o-d transition temperature. This range is higher for odd alkanes than for even alkanes (fig. S4c). This temperature range was comparable to the range over which the endoskeletal droplets vaporized. The vaporization temperature range was quantified by using the standard deviation of the temperature spread for vaporization as seen in fig. S4b. Similar odd/even increasing/decreasing trends in the range of vaporization temperatures can be seen for droplets with pure HC in fig. S4c. This provides further support that the o-d transition to the rotator phase in the solid HC is responsible for the vaporization of the liquid FC phase.

Furthermore, this o-d transition temperature is lower for mixtures of HC than pure components for $C_{20}H_{42}/C_{22}H_{56}$ and $C_{22}H_{46}/C_{24}H_{50}$ mixtures, as seen from their phase diagrams (*23*, *26*, *42*, *43*). Interestingly, the vaporization temperatures observed for FC/HC droplets made with these HC mixtures (plotted in figure 3g) also lies within the rotator phase regions for the phase diagram of the HC only mixtures. Looking at the temperature range, mixtures of HC have a higher range of rotator phase temperatures than the pure components and, similarly, a high range of vaporization temperatures (high standard deviation σ values) is also observed for droplets made with mixtures of HC compared to pure HC (fig. S4d).

Supplementary Movies Captions

Movie S1. Rotating Droplets. $C_5F_{12}/C_{12}F_{26}$ droplets have a remarkable disk-like solid structure inside, which is free to rotate when agitated. The video records this rotation under manual agitation of the glass slide housing the droplet samples, where the flow is laminar, and the velocity of the nearby particle was tracked to be 1.25 μm/s. Slowed down 2X.

Movie S2. Endoskeleton formation. The video shows the formation of the solid disks when a heated liquid droplet is cooled through the transition. The formation of the solid disk is not gradual. Instead, the spherical droplets morph into a non-spherical shape for a while and snap back again into a spherical shape with a solid inside. Slowed down 2.5X.

Movie S3. Vaporization of Sedimentary FC-Enriched Droplet. This video shows vaporization of a single droplet. The endoskeletal droplet shown here consists of $C_5F_{12}/C_{20}H_{42}$ and is stabilized by lipid solution. This spherical droplet contains predominantly C_5F_{12} and hence is spherical and sedimentary. The structure inside is the

solid HC. The droplet formation is sudden and relatively forms a very large vapor bubble owing to the higher amount of volatile component present. Slowed down 2.5X.

Movie S3. Vaporization of Buoyant HC-Enriched Droplet. This video shows vaporization of a single endoskeletal droplet. The droplet shown here consists of $C_5F_{12}/C_{20}H_{42}$ and is stabilized by the hydrocarbon lipid surfactant. The non-spherical droplet contains predominantly $C_{20}H_{42}$ and hence is non-spherical and buoyant. The droplet formation in this case is slow and forms a relatively small vapor bubble owing to the lower amount of volatile component present. As the medium is continually heated, the solid phase melts and forms a lens around the vapor cavity. Slowed down 2.5X.

Movie S5. Optical Vaporization Experiment. Optical microscopy observation of vaporizing $C_5F_{12}/C_{22}H_{46}$ droplets while the sample is being heated. The scale bar is 100 μm. The smallest bubble that we observed after heating in our experiments was 6 μm diameter. Based on the relative molecular volumes of the volatile phase in the vapor and liquid states, we estimate that the droplet diameter that produced such bubbles was about 1.2 μm. Therefore, the size of both the droplet and the bubble are suitable for intravascular applications in biomedical imaging and therapy. Sped up 10X.

Movie S6. Ultrasound Vaporization Experiment. Ultrasound B-mode imaging observation of vaporizing $C_5F_{12}/C_{20}H_{42}$ droplets while the sample is being heated. The bright circular region is the tube wall, and the bright horizontal line below the tube is the thermocouple. The sample is flowing in the tube. The crosshair on the left of the tube denotes the depth where the ultrasound is focused. Sped up 3X.

REFERENCES AND NOTES

1. L. D. Zarzar, V. Sresht, E. M. Sletten, J. A. Kalow, D. Blankschtein, T. M. Swager, Dynamically reconfigurable complex emulsions via tunable interfacial tensions. *Nature* **518**, 520–524 (2015).

2. P. S. Sheeran, P. A. Dayton, Phase-change contrast agents for imaging and therapy. *Curr. Pharm. Des.* **18**, 2152–2165 (2012).

3. L. Shang, Y. Cheng, Y. Zhao, Emerging droplet microfluidics. *Chem. Rev.* **117**, 7964–8040 (2017).

4. H. Peng, D. Zhang, X. Ling, Y. Li, Y. Wang, Q. Yu, X. She, Y. Li, Y. Ding, *n* -alkanes phase change materials and their microencapsulation for thermal energy storage: A critical review. *Energy Fuel* **32**, 7262–7293 (2018).

5. J. Kim, Spray cooling heat transfer: The state of the art. *Int. J. Heat Fluid Flow* **28**, 753–767 (2007).

6. A. B. Theberge, F. Courtois, Y. Schaerli, M. Fischlechner, C. Abell, F. Hollfelder, W. T. S. Huck, Microdroplets in microfluidics: An evolving platform for discoveries in chemistry and biology. *Angew. Chem. Int. Ed.* **49**, 5846–5868 (2010).

7. C. Amole, M. Ardid, I. J. Arnquist, D. M. Asner, D. Baxter, E. Behnke, P. Bhattacharjee, H. Borsodi, M. Bou-Cabo, P. Campion, G. Cao, C. J. Chen, U. Chowdhury, K. Clark, J. I. Collar, P. S. Cooper, M. Crisler, G. Crowder, C. E. Dahl, M. Das, S. Fallows, J. Farine, I. Felis, R. Filgas, F. Girard, G. Giroux, J. Hall, O. Harris, E. W. Hoppe, M. Jin, C. B. Krauss, M. Laurin, I. Lawson, A. Leblanc, I. Levine, W. H. Lippincott, F. Mamedov, D. Maurya, P. Mitra, T. Nania, R. Neilson, A. J. Noble, S. Olson, A. Ortega, A. Plante, R. Podviyanuk, S. Priya, A. E. Robinson, A. Roeder, R. Rucinski, O. Scallon, S. Seth, A. Sonnenschein, N. Starinski, I. Štekl, F. Tardif, E. Vázquez-Jáuregui, J. Wells, U. Wichoski, Y. Yan, V. Zacek, J. Zhang; PICO Collaboration, Dark matter search results from the PICO −60 C_3F_8 bubble chamber. *Phys. Rev. Lett.* **118** (2017).

8. H. Lea-Banks, M. A. O’Reilly, K. Hynynen, Ultrasound-responsive droplets for therapy: A review. *J. Control. Release* **293**, 144–154 (2019).

9. P. A. Mountford, M. A. Borden, On the thermodynamics and kinetics of superheated fluorocarbon phase-change agents. *Adv. Colloid Interf. Sci.* **237**, 15–27 (2016).

10. M. P. Krafft, Fluorocarbons and fluorinated amphiphiles in drug delivery and biomedical research. *Adv. Drug Deliv. Rev.* **47**, 209–228 (2001).
11. O. D. Kripfgans, J. B. Fowlkes, D. L. Miller, O. P. Eldevik, P. L. Carson, Acoustic droplet vaporization for therapeutic and diagnostic applications. *Ultrasound Med. Biol.* **26**, 1177–1189 (2000).
12. P. S. Sheeran, S. H. Luois, L. B. Mullin, T. O. Matsunaga, P. A. Dayton, Design of ultrasonically-activatable nanoparticles using low boiling point perfluorocarbons. *Biomaterials* **33**, 3262–3269 (2012).
13. A. Kabalnov, D. Klein, T. Pelura, E. Schutt, J. Weers, Dissolution of multicomponent microbubbles in the bloodstream: 1. Theory. *Ultrasound Med. Biol.* **24**, 739–749 (1998).
14. A. M. Vezeridis, C. de Gracia Lux, S. A. Barnhill, S. Kim, Z. Wu, S. Jin, J. Lux, N. C. Gianneschi, R. F. Mattrey, Fluorous-phase iron oxide nanoparticles as enhancers of acoustic droplet vaporization of perfluorocarbons with supra-physiologic boiling point. *J. Control. Release* **302**, 54–62 (2019).
15. M. Caggioni, A. V. Bayles, J. Lenis, E. M. Furst, P. T. Spicer, Interfacial stability and shape change of anisotropic endoskeleton droplets. *Soft Matter* **10**, 7647–7652 (2014).
16. M. Caggioni, J. Lenis, A. V. Bayles, E. M. Furst, P. T. Spicer, Temperature-induced collapse, and arrested collapse, of anisotropic endoskeleton droplets. *Langmuir* **31**, 8558–8565 (2015).
17. D. W. Rees Jones, A. J. Wells, Solidification of a disk-shaped crystal from a weakly supercooled binary melt. *Phys. Rev. E* **92**, 022406 (2015).
18. K. A. Dill, S. Bromberg, *Molecular Driving Forces: Statistical Thermodynamics in Biology, Chemistry, Physics, and Nanoscience* (Garland Science, ed. 2, 2011).
19. H. Heinz, T.-J. Lin, R. Kishore Mishra, F. S. Emami, Thermodynamically consistent force fields for the assembly of inorganic, organic, and biological nanostructures: The INTERFACE force field. *Langmuir* **29**, 1754–1765 (2013).
20. J. Zhou, Y. Yang, Y. Yang, D. S. Kim, A. Yuan, X. Tian, C. Ophus, F. Sun, A. K. Schmid, M. Nathanson, H. Heinz, Q. An, H. Zeng, P. Ercius, J. Miao, Observing crystal nucleation in four dimensions using atomic electron tomography. *Nature* **570**, 500–503 (2019).
21. M. Müller, L. G. MacDowell, P. Virnau, K. Binder, Interface properties and bubble nucleation in compressible mixtures containing polymers. *J. Chem. Phys.* **117**, 5480–5496 (2002).

22. J. Chen, E. Zhu, J. Liu, S. Zhang, Z. Lin, X. Duan, H. Heinz, Y. Huang, J. J. De Yoreo, Building two-dimensional materials one row at a time: Avoiding the nucleation barrier. *Science* **362**, 1135–1139 (2018).
23. D. Mondieig, F. Rajabalee, V. Metivaud, H. A. J. Oonk, M. A. Cuevas-Diarte, *n*-Alkane binary molecular alloys. *Chem. Mater.* **16**, 786–798 (2004).
24. A.-J. Briard, M. Bouroukba, D. Petitjean, N. Hubert, M. Dirand, Experimental enthalpy increments from the solid phases to the liquid phase of homologous *n*-alkane series (C_{18} to C_{38} and C_{41}, C_{44}, C_{46}, C_{50}, C_{54}, and C_{60}). *J. Chem. Eng. Data* **48**, 497–513 (2003).
25. N. Denkov, S. Tcholakova, I. Lesov, D. Cholakova, S. K. Smoukov, Self-shaping of oil droplets via the formation of intermediate rotator phases upon cooling. *Nature* **528**, 392–395 (2015).
26. M. Dirand, M. Bouroukba, V. Chevallier, D. Petitjean, E. Behar, V. Ruffier-Meray, Normal alkanes, multialkane synthetic model mixtures, and real petroleum waxes: Crystallographic structures, thermodynamic properties, and crystallization. *J. Chem. Eng. Data* **47**, 115–143 (2002).
27. L. Shang, Y. Cheng, J. Wang, Y. Yu, Y. Zhao, Y. Chen, Z. Gu, Osmotic pressure-triggered cavitation in microcapsules. *Lab Chip* **16**, 251–255 (2016).
28. O. Vincent, P. Marmottant, On the statics and dynamics of fully confined bubbles. *J. Fluid Mech.* **827**, 194–224 (2017).
29. H. Sun, Ab initio calculations and force field development for computer simulation of polysilanes. *Macromolecules* **28**, 701–712 (1995).
30. S. Plimpton, Fast parallel algorithms for short-range molecular dynamics. *J. Comput. Phys.* **117**, 1–19 (1995).
31. J. R. Rumble, Ed., *CRC Handbook of Chemistry and Physics* (CRC Press, Boca Raton, FL, ed. 100, 2019).
32. R. M. Stephenson, S. Malanowski, in *Handbook of the Thermodynamics of Organic Compounds* (Elsevier, New York, 1987), pp. 527–528.
33. P. S. Linstrom, W. G. Mallard, *NIST Chemistry WebBook, NIST Standard Reference Database Number 69* (National Institute of Standards and Technology, Gaithersburg MD, 2018).

34. V. P. Carey, *Liquid-Vapor Phase-Change Phenomena* (Taylor & Francis, New York, ed. 2, 2008).
35. J. Brandrup, E. H. Immergut, E. A. Grulke, *Polymer Handbook* (Wiley, New York, ed. 4, 1999).
36. R. J. Young, P. A. Lovell, *Introduction to Polymers* (CRC Press, Boca Raton, ed. 3, 2011).
37. A. F. M. Barton, *CRC Handbook of Solubility Parameters and Other Cohesion Parameters* (CRC Press, Boca Raton, 1983).
38. J. N. Israelachvili, in *Intermolecular and Surface Forces* (Academic Press, Oxford, ed. 3, 2011), pp. 107–130.
39. P. G. Debenedetti, *Metastable Liquids: Concepts and Principles* (Princeton Univ. Press, Princeton, New Jersey, 1996).
40. C. H. Fisher, Boiling point gives critical temperature. *Chem. Eng.* **96**, 157–158 (1989).
41. P. A. Mountford, A. N. Thomas, M. A. Borden, Thermal activation of superheated lipid-coated perfluorocarbon drops. *Langmuir* **31**, 4627–4634 (2015).
42. Z. Achour, A. Sabour, M. Dirani, M. Hoch, Thermodynamic properties of the *n*-alkanes $C_{19}H_{40}$ to $C_{26}H_{54}$ and their binary phase diagrams. *J. Thermal Anal. Calorim.* **51**, 477–488 (1988).
43. H. Lüth, S. C. Nyburg, P. M. Robinson, H. G. Scott, Crystallographic and calorimetric phase studies of the n-eicosane, $C_{20}H_{42}$:n-docosane, $C_{22}H_{46}$ system. *Mol. Cryst. Liquid Cryst.* **27**, 337–357 (1974).
